# Impact of the Work Environment on Patients’ Safety as Perceived by Nurses in Poland—A Cross-Sectional Study

**DOI:** 10.3390/ijerph182212057

**Published:** 2021-11-17

**Authors:** Iwona Malinowska-Lipień, Agnieszka Micek, Teresa Gabryś, Maria Kózka, Krzysztof Gajda, Agnieszka Gniadek, Tomasz Brzostek, Jason Fletcher, Allison Squires

**Affiliations:** 1 Institute of Nursing and Midwifery, Faculty of Health Sciences, Medical College, Jagiellonian University, 31-501 Krakow, Poland; agnieszka.micek@uj.edu.pl (A.M.); ter.gabrys@uj.edu.pl (T.G.); maria.kozka@uj.edu.pl (M.K.); agnieszka.gniadek@uj.edu.pl (A.G.); tomasz.brzostek@uj.edu.pl (T.B.); 2 Institute of Public Health, Faculty of Health Sciences, Medical College, Jagiellonian University, 31-066 Krakow, Poland; krzysztof.gajda@uj.edu.pl; 3 Rory Meyers College of Nursing, New York University, New York, NY 10012, USA; jason.fletcher@nyu.edu (J.F.); aps6@nyu.edu (A.S.)

**Keywords:** patient safety, work environment, working conditions, workload

## Abstract

Numerous studies have found that organizational features connected with the work environment of nurses have a significant influence on patients’ safety. The aim of this research was to capture nurses’ opinions about patients’ safety and discern relationships with work environment characteristics. This cross-sectional study surveyed 1825 nurses. The research used questionnaire consisting of four parts: (1) covered The Practice Environment Scale of the Nursing Work Index (PES-NWI); (2) assessed the quality of nursing care and care safety; (3) contained information on the most recent duty served by the nurses and (4) captured social and demographic data of participants. The research identified strong association between patient safety assessment and work environment of nurses in the aspect of employment adequacy, cooperation between nurses and doctors, support for nurses from the managing staff, the possibility to participate in the management as well as professional promotion of nurses employed in the hospital (*p* < 0.001). Nurses rated patient safety higher when responsible for a smaller number of patients. Work environment factors such as proper staffing, good cooperation with doctors, support from the management, as well as professional independence are significantly related to nurses’ assessment of patients’ safety.

## 1. Introduction

Patient safety constitutes an important element of the healthcare system and is directly related to the patient’s health, well-being and results of care and cost of treatment. According to Vincent, patient safety means the lack of adverse events resulting from intervention of health care, and not from the patient’s health condition [1]. The World Health Organisation (WHO) defines patient safety as preventing errors and negative effects for patients connected with health care. A 2019 WHO report indicates that one tenth of patients hospitalised in Europe are affected by avoidable adverse events causing suffering and losses for the patients, their families as well as health care providers [2]. Other studies show that even half of adverse events could be prevented [3,4]. In 2009 the list of six international patient safety goals was introduced, covering correct identification of the patient, clear and effective communication, safety in administering medications, increasing safety of operations, decreasing risk of infections, decreasing the occurrence of falls [5].

Nurses are the largest professional group in the health care system and the most direct contact with the hospitalized patient; thus nursing care is one of the areas of health services that significantly influence patient safety [6]. Nursing services are concerned with patients’ perceptions of the quality of services provided by nurses while in hospital. Nursing care is also considered one of the most important features of the healthcare sector. Scientists made significant discoveries regarding the interaction between patient outcomes and nursing services. They highlighted the strong relationship between nursing services and services provided to patients [7,8] Since the quality of patient safety is affected by how an organization manages threats to it, where a nurse works is an important factor that influences how nurses are able to mitigate threats to patient safety. Thus, the work environment of nurses significantly influences professional nursing practice [9]. The results of the presented research may have an impact on the improvement of patient safety, provided that the management is open to the prospects linking patient safety with the working conditions of nurses. Numerous studies show that organisational features of nurses’ work environment have a significant influence on patients’ safety [6,10,11,12]. Aiken et al. highlighted the necessity of creating an optimal work environment for nurses [13]. Other authors point out that creating an optimal work environment requires both ward and hospital level assessments to identify strengths and areas in need of improvement [14,15,16].

Hospitals in Poland and around the world face unprecedented changes in care delivery, ranging from increased complexity to resource challenges and workforce shortages. Any vulnerabilities in an organization were made clear by the COVID-19 pandemic, including threats to patient safety. An additional factor influencing the functioning of health care is demographic changes, which result in the increase of elderly people, chronically ill and requiring frequent hospitalisations, as well as aging staff with limited younger replacements [17]. When it comes to adjusting to the changing character of work in hospitals, the management and direct superiors play an important role as leaders [11,17]. In work environments where the issues of patient safety are consistently addressed, the leadership might have a positive influence on the staff’s efficiency, the level of their awareness, as well as increasing engagement in the improvement of care quality and safety [11,17,18].

Evaluations of nurses’ work environments are recommended as an effective strategy to improve the work environment and limit professional burnout of nurses [19]. Research carried out in the USA in “hospitals attracting nursing staff and patients”, so called “magnet hospitals”, showed that good nursing practices are associated with excellent quality of care and positive results both for patients and for nurses [20]. Magnet hospitals” promote high quality care over patients, safety, interdisciplinary professional relations, positive communication, professional care models, greater possibilities of professional development and better working conditions more successfully attract and retain specialists than other hospitals [21]. They also regularly survey nursing staff for their opinions about the work environment and to identify potential threats to patient safety observed by nurses.

Using the aforementioned strategy, the purpose of this study was to survey nurses about their perceptions of the relationship between work environment characteristics and patient safety in Polish hospitals. The goal of the study was to determine key opportunities where hospitals could direct efforts to improve patient safety and nurses’ work environments simultaneously.

## 2. Materials and Methods

This cross-sectional national study surveyed female and male nurses employed in surgical and internal wards in 21 randomly chosen hospitals in Poland. Only state funded hospitals were included in the study. This research took place between 2018–2019 and was carried out according to the baseline of the RN4CAST project funded by 7th European Union Framework Program between 2009–2011, in which Poland participated as one of the twelve EU states [22].

### 2.1. Sample

Nurses employed in one of the 21 hospitals who had worked there for at least six months were invited to participate in the study. Students and administrators were excluded from the study.

### 2.2. Instruments

The same research protocol and measures used in RN4CAST were used to collect the data in this study. These methods and measures have been extensively validated and described in published research papers [20].

The entire survey consisted of four parts. The first part contained The Practice Environment Scale of the Nursing Work Index (PES-NWI). The PES-NWI consists of 32 Likert type questions (1: “Strongly Disagree” --> 4 “Strongly Agree”) including five sub-scales: Staffing and resource adequacy (four questions); Cooperation in therapeutic team (seven questions); Support for nurses from management (four questions); Nurses’ participation in hospital management (eight questions); Support for providing high standards of quality of nursing care (nine questions). In this study, mean scores for each of the five subscales were calculated and used. Higher scores meant a more favorable working environment. This part also contained nine questions concerning overall assessment of working conditions and satisfaction with work in hospital in the aspect of: flexibility of scheduling working time, independence at work, professional status, satisfaction with remuneration, possibility of promotion, training, obtaining yearly and training holidays, use of sick leave. In these questions, the respondents marked their answers on a 4-point scale (from 1—very unsatisfied to 4—very satisfied), provided with each statement. The responses were grouped into three categories, i.e., unsatisfied made up of very unsatisfied and a little unsatisfied, medium satisfied and very satisfied. The second part of the questionnaire listed questions concerning patient safety assessment and nursing care quality. The third part contained information on the most recent duty served by the nurses. This part of the questionnaire was used to assess workload, uncertified staff’s participation in care and information on the activities which nurses did not manage to perform during their shift due to the lack of time. In this part respondents were asked to determine the number of patients subjected to direct care and requiring frequent monitoring of life activities, as well as whether workload during the most recent shift was representative for the position of the nurses questioned. The fourth part of the questionnaire contained social and demographic data. Nurses were asked to provide gender, age and work experience data [22].

### 2.3. Data Collection

The research was carried out using the method of diagnostic polling with an anonymous survey independently completed by nurses. Nurses were informed about the aim and rules of the research and agreed to participate in it. The research was performed according to the rules of The Declaration of Helsinki and was also carried out with the permission from the Bioethics Commission of Jagiellonian University No. 1072.6120.111.2018.

### 2.4. Statistical Analysis

The statistical analysis was performed with R software version 3.6.1 (Development Core Team, Vienna, Austria). Compatibility with normal distribution for quantitative variables was checked using the Shapiro-Wilk test, and homogeneity of variance was assessed using Levene’s test, correspondingly when two or more variables were being compared. Descriptive statistics were reported using medians (Me) and inter-quartile range (Q_1_–Q_3_). Qualitative variables were characterised presenting the number of cases (n) and percentage (%). In analyses comparing a quantitative variable between groups, non-parametric Mann-Whitney U–test or Kruskal-Wallis sum rank test were employed. Association between qualitative variables was determined using the Chi-square test for independence. For all analyses, significance level *α* = 0.05 was used.

The *dependent variable* represented nurses’ opinion on: work environment, level of satisfaction with various aspects connected to the performed work, workload, care quality, occurrence of adverse events (incorrect administration of medication, bedsores, falls, infections), necessary but not performed care activities, nurses’ sense of security at workplace.

The *independent variable* was nurses’ rating of patient safety measured with the question: “Please provide an overall assessment of your ward in the matter of patients’ safety”. The question contained five possible answers: unsatisfactory, poor, acceptable, very good and excellent. The answers were sorted to create three categories, i.e., low assessment created from answers “unsatisfactory” and “poor”, middle assessment corresponding to “acceptable”, and high assessment created from “very good” and “excellent”.

## 3. Results

A total of 1825 nurses (1762 females, 97.5%) participated in the study, representing 0.8% of professionally active Polish nurses. Nurses’ average age was 44.15 years (SD = 10.27). The youngest participant of the survey was 22 years old, and the oldest 69 years old. Participants had worked as nurses for 21.89 years on average (SD = 11.39), ranging from 0.5 years to 50 years. The average length of service on the nursing position in the hospital where a nurse was employed at the time of the research was 17.85 years (SD = 11.76). Among responding nurses, 498 (27.3%) assessed patients’ safety in the ward where they worked at a high level, 926 (50.7%) at a medium level, while 401 (22.0%) at a low level.

**Figure 1:**
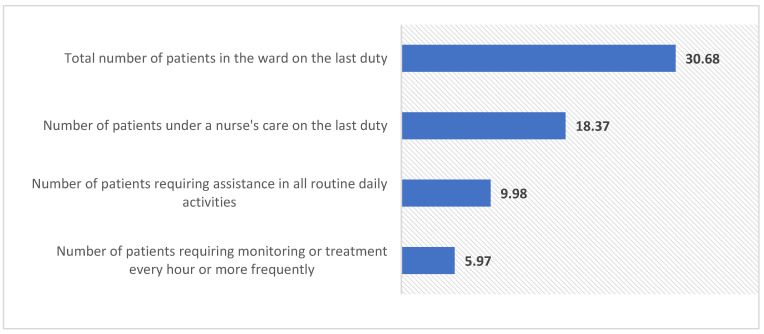
The characteristics of workload based on the number of patients under care during the most recent shift. Labels in the image represent median values.

During their most recent shift in the ward there were 30.68 patients on average (SD = 13.01), nurses were directly responsible for 18.37 patients on average (SD = 11.28), 9.98 patients on average required assistance in all daily activities (SD = 7.6), and 5.97 patients on average required monitoring or treatments every hour or more frequently (SD = 5.64). The workload characteristics based on the number of patients under care during the most recent shift is presented in Figure 1.

### 3.1. Patient Safety and Working Conditions

Amongst the respondents, 65.4% of the surveyed group rated working conditions as excellent, 32.1% of as good, and 12.4% in the group declaring an unsatisfactory grade. The analysis of the research showed that there was an association between overall assessment of working conditions and overall assessment of safety (*p* < 0.001). In the opinion of nurses, higher positive ratings of working conditions in the hospital corresponded to higher ratings of overall patient safety.

Overall assessment of patient safety in the ward was related to changes in the quality of care in the last year (*p* < 0.001). Nurses reporting an improvement of care quality in the hospital in the year reported higher levels of patient safety. Ratings indicating an improvement in patient safety during the last year were received from 58.7% of nurses. Simultaneously, 25.5% indicated no improvement and 8.1% who reported a decrease of patient safety.

The analysis of The Practice Environment Scale of the Nursing Work Index (PES-NWI) characteristics showed that the higher nurses rated patient safety, the higher they rated adequacy of human and material resources, cooperation between nurses and doctors, support for nurses from the managing staff, possibility for nurses’ participation in managing the hospital, and support for providing high standards of nursing care quality (*p* < 0.001); Table 1.

### 3.2. Patient Safety and Nurses’ Satisfaction with Autonomy and Professional Development

The lower the nurses assessed the patient’s safety in the ward, the lower they assessed their satisfaction with autonomy and professional development. It was shown that 71.2% of the low-safety group were unsatisfied with the possibility of promotion, compared to the medium–56.3% and high-safety group–46.0%. Moreover, 59.2% of the respondents who rated safety as low reported unsatisfaction with independence at work (autonomous practice) compared to the group with medium–38.4% and high safety level–20.2%; Table 2.

### 3.3. Patients’ Safety and Nurses’ Sense of Safety

Multiple relationships were revealed between a nurse’s assessment of patient safety in the ward and nurses’ opinion on safe working conditions. Notable relationships included good flow of information between providers and culture of awareness that when errors occur, preventable event discussions happen to determine how to prevent them in the future. Organizations with non-punitive environments also received higher ratings by nurses on patient safety overall (*p* < 0.001).

By contrast, nurses who perceived themselves as working in punitive environments were less likely to rate patient safety well. Approximately 27% of nurses reported that they are severely punished for their errors without forgiveness. The analysis further showed that the more frequently nurses declared that they are allowed to question the decisions or actions of their supervisors, the higher they rated patient safety. The analysis further showed that the nurses assessed the patient’s safety lower, the more they declared that they were unable to question the decisions or actions of their superiors. Table 3 illustrates how across all measures, ratings were statistically significantly different.

### 3.4. Patients’ Safety and Nurses’ Workload

Workload, including time spent working beyond scheduled shift hours, also affected patient safety ratings. Nurses who were working during their most recent shift beyond their contractual time of work assessed safety lower than other nurses who were working according to their contracted hours; low assessment was provided respectively by 19.9% vs. 32.5%; *p* < 0.001.

Nurse-to-patient ratios also affected their perception of patient safety. The higher the nurses assessed overall patients’ safety, the lower the number of patients per nurse, the (medians of the indicator of patients per nurse were respectively: 1.71, 1.62 and 1.55 in the subsequently higher assessment groups for safety (*p* = 0.041).

The surveyed nurses who during their most recent shift declared that they cared for a higher number of patients than usual more frequently assessed safety lower than other nurses; low assessment was declared by 28.1% of nurses who cared for a larger number of patients, 19.0% of those caring for the same number of patients, and 20.9% of those caring for a smaller number of patients during their most recent shift, respectively (*p* < 0.001).

Nurses’ assessment of patient safety in the ward was also connected with the number and health condition of the patients for whom they cared during their most recent shift. Nurses who cared on average for a smaller number of patients requiring assistance in all routine daily activities as well as frequent monitoring of their health condition assessed safety in the ward higher; *p* < 0.001; Table 4.

### 3.5. Patients’ Safety and Occurrence of Adverse Events, Failure to Perform Necessary Activities and Care Quality

Ward safety assessments were associated with the occurrence of adverse events associated with the quality of nursing care, namely administration of a wrong medication, at a wrong time or in an incorrect dosage, bedsores, falls, and infections. Higher assessment of safety in the ward were associated with less frequent declaration of such events (*p* < 0.001).

Patient safety assessment in the ward was related to nurses performing activities related to direct patient care (*p* < 0.001), performance of activity was more often declared by those who assessed safety as high, especially in the range of delivered services such as monitoring of a patient according to the rules (respectively 85.2.% vs. 62.0%), care over the patient’s skin (respectively 80.9% vs. 48.1%), relieving the patient’s pain (respectively 95.1% vs. 81.8%), administering medication on time (respectively 85.4% vs. 65.3%), proper recording of nursing care (respectively 88.4% vs. 58.2%); Table 5.

## 4. Discussion

Thanks to their constant contacts with patients, nurses constitute a good source of information for safety assessment [23]. In the healthcare sector in Poland, over the last twenty years, a decline in the employment of nurses in relation to the growing demand for care has been recorded. The low staffing levels adversely affect the functioning of the whole health care system, and thus the health safety of Polish society. According to the data obtained from the Council of Nurses and Midwives (Polish: NRPiP), the current average age of nurses in Poland is 52.59 years [24]. According to the Central Register of Nurses, as many as 40,000 nurses are over the age of 56. This means that this group will be eligible for retirement within the next 4 years, as in Poland women can retire at the age of 60 and men at 67 [24], although it is often the case that nurses of retirement age continue to work as nurses. The current research determined that the higher working conditions in a hospital were rated, the higher patients’ safety was reported by nurses. The percentage values of high ratings were: 65.4%, 32.1%, and 12.4% in the groups of nurses declaring that working conditions are excellent, good and unsatisfactory, respectively. Research by other authors confirms that a positive work environment of nurses, including working conditions and specific features of the ward significantly influence work satisfaction, engagement and care quality [10,25,26].

The analysis of features of nurses’ practice environment (PES-NWI) indicated that the higher the human and material resources adequacy, cooperation between nurses and doctors, support received by nurses from management and the possibility for nurses’ participation in hospital management reported, the higher the patient safety assessed in the hospital was. In a therapeutic team, where doctors and nurses play a key role, it is important and necessary to elaborate rules for cooperation between them based on trust, respect, good communication and familiarity with competences aimed at safety and care quality. Clarke showed that good nurse-doctor relationships, communication and sufficient staffing of nurses have an impact on the less frequent occurrence of adverse events [27]. According to Ajeigbe et al., active nurse practice where one works in a team increases job satisfaction, improves care quality and efficiency [28].

Apart from good relations within a team, is essential to grant nurses practical autonomy as well as support from the managing staff. These features are often connected with workers’ job satisfaction and low level of intention of leaving a position [29]. Our research showed that the higher nurses’ satisfaction with a possibility of promotion and their sense of professional independence was, the higher they rated patients’ safety in the ward.

In our research, actions and attitudes of the hospital management had a significant connection with patients’ safety assessment. The higher priority of patient safety for the managing staff was related to the higher assessment of patients’ safety by nurses. However, if measures to prevent further errors were not discussed on the ward level, nurses more frequently assessed patient safety at the low level.

According to Duffield et al., support from management is a key factor of positive work environment which promotes the development of nursing practice and patients’ safety. Manager traits such as availability, participation in making decisions related to an entity, as well as flexibility and support given to subordinates are in line with the increase of satisfaction with work, greater retention of qualified specialists and lower willingness of the staff to quit jobs [30].

Another important finding was that nearly one third (32.5%) of nurses who worked beyond their contracted time assessed safety as low, while a similar percentage value in the group of nurses working according to their work time was significantly lower (19.9%). Our research did not include either a weekly work time of nurses, or the reason for overtime, or the frequency of serving shifts in a particular hospital by nurses beyond contracted time. From the perspective of safety assessment, these issues could explain low safety ratings associated with working overtime. Other research has found that working in the range of 12 h or more a day on the position of a nurse in a hospital is related to adverse effects on patients’ safety and decrease of care quality [31,32]. Work time beyond the normal shift causes tiredness and lower vigilance on the part of the nurse, which may result in the increase of adverse events, lower work efficiency, tendency to omit some duties or it may lead to interferences in decision making [32].

According to Hollnagel et al., studies on patients’ safety ought to pay attention to factors focused not only on errors but measures to increase health care safety. Such attitude allows to understand and learn more about the complexity of health care and challenges which health care workers face on a daily basis, trying not to expose patients to threats [33]. The results of own research point to the correlation between nurses’ opinion concerning patients’ safety with the features of their work environment. The results of this research may have an influence on the improvement in patients’ safety provided that the managing staffs show openness to the perspectives bonding patients’ safety with nurses’ working conditions.

## 5. Limitations

The study is not without limitations. It is a cross-sectional study, but data were collected only from surgical and internal wards. Therefore, the results of this study are limited to the answers provided by nurses working in the selected specificity and cannot be generalized to all hospital wards in Poland. Secondly, the assessment of patient safety and the working environment is a subjective opinion of nurses. In subsequent studies, the study should be expanded to include patients’ opinions on safety and management’s opinions on the work environment. Third, nurses working in only 21 hospitals in Poland participated in the study. Poland is divided into 16 voivodships and from each voivodeship nursing staff from at least one hospital took part in the study. Despite this, the results from these 21 hospitals cannot reflect the situation of all hospitals in Poland.

## 6. Conclusions

Work environment factors such as proper staffing, good cooperation with doctors, support from the management, as well as professional independence are significantly related to nurses’ assessment of patients’ safety.

## Figures and Tables

**Table 1:** Characteristics of work environment (PES-NWI) across overall patient safety assessment.

Patient Safety and Work Environment (PES-NWI)	Overall Patient Safety Assessment			
	Low	Medium	High	
	Q2 (Q1–Q3)	Q2 (Q1–Q3)	Q2 (Q1–Q3)	*p*
Staffing and resource adequacy	1.5 (1.0–1.75)	1.75 (1.25–2.25)	2 (1.5–2.5)	<0.001
Cooperation in Therapeutic team	1.75 (1.38–2.25)	2.25 (1.88–2.75)	2.5 (2.0–2.88)	<0.001
Support for nurses from management	2 (1.5–2.62)	2.5 (2.0–3.0)	3 (2.5–3.5)	<0.001
Nurses’ participation in hospital management	2 (1.43–2.43)	2.43 (2.0–2.86)	2.71 (2.29–3.0)	<0.001
Support for providing high Standards of quality of nursing care	2.33 (1.94–2.78)	2.78 (2.44–3.11)	3.11 (2.67–3.44)	<0.001

Note: Q2—median, Q1–Q3—interquartile range; *p*-value; the results of Kruskal-Wallis sum rank test.

**Table 2:** Safety and nurses’ satisfaction with various aspects of work.

Nurses’ Level of Satisfaction with Work:	Overall Assessment of Patients’ Safety			
Flexibility of Work Schedule	Low	Medium	High	*p*
	n (%)	n (%)	n (%)	
unsatisfied	192 (48.7)	255 (27.9)	72 (14.7)	<0.001
medium satisfied	173 (43.9)	501 (54.9)	273 (55.8)	
very satisfied	29 (7.4)	157 (17.2)	144 (29.4)	
**Possibility of Promotion**				
unsatisfied	279 (71.2)	507 (56.3)	224 (46.0)	<0.001
medium satisfied	105 (26.8)	367 (40.7)	216 (44.4)	
very satisfied	8 (2.0)	27 (3.0)	47 (9.7)	
**Independence at Work**				
unsatisfied	232 (59.2)	349 (38.4)	98 (20.2)	<0.001
medium satisfied	149 (38)	497 (54.6)	280 (57.7)	
very satisfied	11 (2.8)	64 (7.0)	107 (22.1)	
**Professional Status**				
unsatisfied	236 (60.4)	370 (41.2)	118 (24.9)	<0.001
medium satisfied	137 (35)	471 (52.4)	261 (55.1)	
very satisfied	18 (4.6)	57 (6.3)	95 (20.0)	
**Satisfaction with Remuneration**				
unsatisfied	294 (74.2)	584 (63.5)	266 (54.4)	<0.001
medium satisfied	91 (23.0)	304 (33.1)	184 (37.6)	
very satisfied	11 (2.8)	31 (3.4)	39 (8.0)	
**Possibility of Training**				
unsatisfied	252 (63.8)	361 (39.6)	133 (27.3)	<0.001
medium satisfied	129 (32.7)	456 (50.0)	227 (46.5)	
very satisfied	14 (3.5)	95 (10.4)	128 (26.2)	
**Yearly Holidays**				
unsatisfied	285 (72.9)	479 (53.6)	199 (41.3)	<0.001
medium satisfied	88 (22.5)	339 (37.9)	187 (38.8)	
very satisfied	18 (4.6)	76 (8.5)	96 (19.9)	
**Sick Leaves**				
unsatisfied	248 (63.9)	389 (43.7)	162 (34.5)	<0.001
medium satisfied	125 (32.2)	429 (48.1)	227 (48.3)	
very satisfied	15 (3.9)	73 (8.2)	81 (17.2)	
**Training Leaves**				
unsatisfied	309 (79.2)	537 (60.5)	251 (52.3)	<0.001
medium satisfied	69 (17.7)	276 (31.1)	153 (31.9)	
very satisfied	12 (3.1)	74 (8.3)	76 (15.8)	

Note: *p*-value; the results of Chi-square test.

**Table 3:** Patients’ safety and nurses’ sense of safety.

Sense of Safety	Overall Assessment of Patients’ Safety			
Staff Feel That They Are Severely Punished without Forgiveness for Their Errors	Low	Medium	High	*p*
	n (%)	n (%)	n (%)	
I do not agree	143 (35.7)	374 (40.4)	246 (49.4)	<0.001
I have no opinion	150 (37.4)	414 (44.7)	181 (36.3)	
I agree	108 (26.9)	138 (14.9)	71 (14.3)	
**Important Information on a Patient is Often Lost during Shift Change**				
I do not agree	224 (55.9)	618 (66.7)	407 (81.7)	<0.001
I have no opinion	89 (22.2)	195 (21.1)	53 (10.6)	
I agree	88 (21.9)	113 (12.2)	38 (7.6)	
**Things “Disappear somewhere” while a Patient is being Moved from One Ward to Another**				
I do not agree	299 (74.6)	723 (78.1)	437 (87.8)	<0.001
I have no opinion	63 (15.7)	156 (16.8)	41 (8.2)	
I agree	39 (9.7)	47 (5.1)	20 (4.0)	
**Staff Feel they can Question Supervisors’ Decisions or Actions**				
I do not agree	273 (68.1)	508 (54.9)	241 (48.4)	<0.001
I have no opinion	87 (21.7)	330 (35.6)	174 (34.9)	
I agree	41 (10.2)	88 (9.5)	83 (16.7)	
**In The Ward It Is Discussed How To Prevent Further Errors**				
I do not agree	174 (43.4)	185 (20.0)	55 (11.0)	<0.001
I have no opinion	100 (24.9)	233 (25.2)	63 (12.7)	
I agree	127 (31.7)	508 (54.9)	380 (76.3)	
**Staffs are Informed about Changes Implemented Based on Reports about Committed Negligence and Errors.**				
I do not agree	143 (35.7)	168 (18.1)	49 (9.8)	<0.001
I have no opinion	101 (25.2)	215 (23.2)	76 (15.3)	
I agree	157 (39.2)	543 (58.6)	373 (74.9)	
**Actions of Hospital’s Management Show that Patient Safety is a Priority Issue**				
I do not agree	254 (63.3)	278 (30.0)	76 (15.3)	<0.001
I have no opinion	106 (26.4)	364 (39.3)	140 (28.1)	
I agree	41 (10.2)	284 (30.7)	282 (56.6)	

Note: *p*-value; the results of Chi-square test.

**Table 4:** Patients’ safety and nurses’ workload during their most recent shift.

During the Most Recent Shift:	Overall Patient Safety Assessment			
	Low	Medium	High	*p*
	Q2 (Q1–Q3)	Q2 (Q1–Q3)	Q2 (Q1–Q3)	
Number of patients for whom a nurse is responsible	20 (12–28)	15 (10–25)	12 (8–22)	<0.001
Number of patients requiring assistance in routine daily activities	10 (6–16)	8 (5–12)	6 (4–10)	<0.001
Number of patients requiring monitoring or treatment every hour or more frequently	5 (3–10)	4 (2–7)	4 (2–8)	<0.001
Total number of patients in the ward	30 (24–40)	30 (23–40)	27 (20–35)	<0.001
Total number of nurses providing direct care over patients	3 (2–4)	3 (3–4)	3 (3–4)	0.054
Total number of other staff providing direct care over patients during the most recent shift	1 (0–3)	1.5 (1–3)	2 (1–3)	0.545

Note: Q2—median, Q1–Q3—interquartile range; *p*-value; the results of Kruskal-Wallis sum rank test.

**Table 5:** Patients’ safety and occurrence of adverse events and unperformed nursing activities.

Frequency of Occurrence of Adverse Events	Overall Assessment of Patients’ Safety			
A Patient Received Wrong Medication at a Wrong Time or in a Wrong Dosage	Low	Medium	High	*p*
	n (%)	n (%)	n (%)	
never	128 (32.9)	373 (41.2)	284 (57.7)	<0.001
rarely	174 (44.7)	427 (47.1)	187 (38.0)	
often	87 (22.4)	106 (11.7)	21 (4.3)	
**Bedsores Occurred after Admission**				
never	35 (8.9)	55 (6.1)	55 (11.2)	<0.001
rarely	192 (48.9)	536 (59.4)	328 (66.8)	
often	166 (42.2)	311 (34.5)	108 (22.0)	
**Patient’s Fall with Injury**				
never	35 (9.0)	78 (8.7)	83 (16.9)	<0.001
rarely	219 (56.6)	611 (68.3)	356 (72.7)	
often	133 (34.4)	205 (22.9)	51 (10.4)	
**Infection Related to Care Occurred-Urinary Tract Infection**				
never	50 (12.9)	151 (16.9)	124 (25.3)	<0.001
rarely	219 (56.6)	538 (60.4)	306 (62.4)	
often	118 (30.5)	202 (22.7)	60 (12.2)	
**Infection Related to Care Occurred-Blood Infection**				
never	118 (30.6)	322 (36.5)	239 (50.2)	<0.001
rarely	181 (47.0)	441 (50.0)	207 (43.5)	
often	86 (22.3)	119 (13.5)	30 (6.3)	
**Infection Related to Care Occurred-Pneumonia**				
never	58 (15.2)	162 (18.3)	132 (27.3)	<0.001
rarely	210 (55.0)	545 (61.6)	297 (61.5)	
often	114 (29.8)	178 (20.1)	54 (11.2)	
**Activities which were not Performed during the most Recent Shift due to Lack of Time**				
**According to the Rules of Monitoring a Patient**				
performed	245 (62.0)	750 (81.7)	419 (85.2)	<0.001
unperformed	150 (38.0)	168 (18.3)	73 (14.8)	
**Skin Care**				
performed	190 (48.1)	675 (73.7)	398 (80.9)	<0.001
unperformed	205 (51.9)	241 (26.3)	94 (19.1)	
**Oral Hygiene**				
performed	133 (33.7)	443 (48.3)	294 (59.8)	<0.001
unperformed	262 (66.3)	475 (51.7)	198 (40.2)	
**Pain Alleviation**				
performed	323 (81.8)	851 (92.8)	468 (95.1)	<0.001
unperformed	72 (18.2)	66 (7.2)	24 (4.9)	
**Calming/Conversation with Patients**				
performed	152 (38.5)	517 (56.3)	330 (67.1)	<0.001
unperformed	243 (61.5)	401 (43.7)	162 (32.9)	
**Patients’ and their Families’ Education**				
performed	99 (25.1)	323 (35.2)	238 (48.4)	<0.001
unperformed	296 (74.9)	595 (64.8)	254 (51.6)	
**Treatment and Procedures**				
performed	332 (84.1)	854 (93.0)	476 (96.7)	<0.001
unperformed	63 (15.9)	64 (7.0)	16 (3.3)	
**Administering Medication on time**				
performed	258 (65.3)	731 (79.8)	420 (85.4)	<0.001
unperformed	137 (34.7)	185 (20.2)	72 (14.6)	
**Preparation of Patients and their Families for Discharge**				
performed	221 (55.9)	614 (66.9)	365 (74.2)	<0.001
unperformed	174 (44.1)	304 (33.1)	127 (25.8)	
**Correct Recording of Nursing Care**				
performed	230 (58.2)	713 (77.7)	435 (88.4)	<0.001
unperformed	165 (41.8)	205 (22.3)	57 (11.6)	
**Creating or Updating Nursing Care Plan/Guidelines for Nursing care**				
performed	189 (47.8)	554 (60.3)	356 (72.4)	<0.001
unperformed	206 (52.2)	364 (39.7)	136 (27.6)	
**Care Planning**				
performed	180 (45.6)	580 (63.2)	354 (72.0)	<0.001
unperformed	215 (54.4)	338 (36.8)	138 (28.0)	
**Frequent Change of s Patient’s Position**				
performed	153 (38.7)	499 (54.4)	354 (72.0)	<0.001
unperformed	242 (61.3)	419 (45.6)	138 (28.0)	

Note: *p*-value; the results of Chi-square test.
